# Enabling artificial placenta cannulation: mechanisms of postnatal umbilical vessel closure and strategies to prolong patency

**DOI:** 10.3389/fphys.2026.1928360

**Published:** 2026-09-21

**Authors:** Nils B. Römer, Sjoerd Haalstra, Danny J. M. van Galen, Daan R. M. G. Ophelders, G. Adrienne Weiss, Jan Däbritz, Tim G. A. M. Wolfs, Jutta Arens, Christoph Fusch, Niels Rochow

**Affiliations:** 1 Department of Pediatrics, Paracelsus Medical University, Nürnberg, Germany; 2 Faculty of Medicine, University of Lübeck, Lübeck, Germany; 3 Department of Pediatrics, Greifswald University Medical Center, Greifswald, Germany; 4 Department of Biomechanical Engineering, Engineering Organ Support Technologies, TechMed Centre, University of Twente, Enschede, Netherlands; 5 Department of Pediatrics, MosaKids Children’s Hospital, Maastricht University Medical Centre, Maastricht, Netherlands; 6 GROW Research Institute for Oncology and Reproduction, Maastricht University, Maastricht, Netherlands; 7 Department of Pediatrics, Klinikum Ernst von Bergmann, Potsdam, Germany; 8 Institute for Clinical Research and Systems Medicine, HMU Health and Medical University, Potsdam, Germany; 9 Department of Pediatrics, McMaster University, Hamilton, ON, Canada; 10 Department of Pediatrics, Rostock University Medical Center, Rostock, Germany

**Keywords:** artificial womb, large-bore cannulation, serotonin (5-HT), thromboxane A2 (TxA2), umbilical artery, umbilical catheter, umbilical vein, vasoconstriction

## Abstract

Umbilical vessel closure after birth prevents hemorrhage but limits the time window for large-bore umbilical cannulation required for artificial placenta support. This review synthesizes physiologic triggers, vasoactive pathways, and vessel-wall specializations that drive rapid postnatal occlusion and highlights engineering-relevant targets to prolong functional patency. Umbilical vessel closure is promoted by the transition to extrauterine life (cooling, rising oxygen tension, oxidative stress, loss of placental vasodilator influence), altered hemodynamics after lung aeration, and mechanical stimulation of the cord. At the molecular level, vasoconstriction is driven by redundant mediator networks—most prominently serotonin and thromboxane A_2_, with supportive contributions from endothelin-1, acetylcholine, bradykinin, catecholamines, and histamine—occurring in the context of limited vasorelaxation capacity. Structurally, the umbilical arteries exhibit a distinctive bilayered tunica media: an outer contractile smooth-muscle-rich layer and an inner proteoglycan-rich layer prone to inward buckling, supported by longitudinal intimal protrusions (Folds of Hoboken) that further promote mechanical occlusion of the lumen. Together, these features form a robust “fail-safe” closure program, although the specific mechanisms governing the intra-abdominal segments remain less understood.

## Introduction

Research on artificial placenta and artificial womb technologies aims to support neonates with severe respiratory or renal failure (van Galen et al., 2025). These systems require large-bore vascular access, ideally via the umbilical vessels. However, after birth, vasoconstriction of umbilical vessels occurs, umbilico-placental blood flow ceases, and vascular remodelling begins. The long distal segments of the umbilical arteries fibrose to form the paired medial umbilical ligaments, while short proximal segments remain patent giving rise to the superior vesical arteries. The umbilical vein and ductus venosus subsequently obliterate, becoming the ligamentum teres hepatis and ligamentum venosum, respectively.

Large-bore access can be obtained by cannulation of the native umbilical vessels while the neonate remains attached to the placenta and fetal circulation persists. Once the cord is clamped, access requires deliberate re-opening of the umbilical vessels to re-establish patency, and the postnatal window for this is very brief.

However, during the first hours after birth, many neonates who ultimately will need artificial placenta support cannot be identified. This is due to the fact that only about 25% of the neonates that require mechanical ventilation and thus would be eligible for treatment with an artificial placenta become clinically apparent within the first 3 hours postpartum (Moya et al., 2019). Consequently, large-bore access may have to be established in umbilical vessels at different stages of postnatal vasoconstriction and remodelling, which entails more invasive techniques and higher risks of vascular injury and bleeding.

*In utero*, the vessel diameter and the umbilical blood flow are dependent on the gestational age and increase during pregnancy (Oriji et al., 2022). Umbilical arterial and venous diameters increase from approximately 2.7 mm and 4.9 mm at 20 weeks’ gestation to about 4.6 mm and 9.1 mm at term (40 weeks) (Barbieri et al., 2012). This requires different cannula sizes for the artificial placenta, dependent on the gestational age. Furthermore, the cannula wall reduces the effective inner diameter in comparison to the native *in utero* vessel lumen. However, smaller cannulation diameters may be sufficient. While the natural placenta provides full systemic support *in utero*, an artificial placenta typically serves as a lung- and kidney-assist device. Therefore, complete multi-organ support with the same diameters as *in utero* is generally not required during artificial placenta application (Schoberer et al., 2014; van Galen et al., 2025).

Accordingly, the physiological mechanisms governing postnatal umbilical vessel constriction and patency must be understood to enable reliable large-bore vascular access. After birth, blood flow between the newborn and the placenta ceases spontaneously within minutes - even without immediate cord clamping - as the umbilical arteries constrict and, subsequently, the vein passively closes. This vasoconstriction is driven by multiple physiological triggers, some of which may be amenable to modulation. Postnatal vascular occlusion appears to result from a synergistic interaction between vasoactive mediators and mechanical factors acting on a uniquely structured vessel wall, which closes the lumen rapidly, completely and irreversibly.

This narrative review aims to (i) delineate the mechanisms of postnatal umbilical vessel closure; (ii) distinguish the intra-abdominal (intracorporeal) from the extra-abdominal (cord and placental) vessel segments, which differ in their regulation; and (iii) derive strategies to maintain umbilical vessel patency and, where necessary, to achieve controlled re-opening, so that large-bore access for an artificial placenta can be established. The overarching objective is to extend the practical cannulation window during the critical postnatal decision-making period while minimising the risk of vascular injury and bleeding during vessel dilation.

## Methods

A literature search was performed using PubMed, Google Scholar and Web of Science. In addition, the reference lists of eligible articles were screened.

The initial search strategy comprised topic-specific keywords such as “umbilical vessel closure” and “umbilical vessel constriction”.

After an initial screening of potential vasoactive substances and physiological mechanisms, a more detailed search was performed to identify further articles for inclusion.

Peer-reviewed original research articles published in German or English were eligible for inclusion, whereas studies that did not meet accepted standards of scientific quality were excluded.

No restrictions were applied regarding the year of publication. Most fundamental research studies were conducted from 1970 to 2010, whereas in recent years a shift towards clinical studies (pregnancy comorbidities related to reduced placental blood flow) has been observed.

## Anatomy

The umbilical cord contains two arteries and a single vein. The umbilical arteries carry deoxygenated, waste-laden fetal blood from the fetus to the placenta. In the placenta, the fetal blood is oxygenated, metabolic waste products are eliminated, and nutrients are delivered. After placental exchange, the blood returns to the fetus via the umbilical vein. Anatomically, the umbilical vessels are divided by the umbilical ring (a fascial aperture in the abdominal wall) into intra-abdominal and extra-abdominal segments.

Intra-abdominally, the umbilical arteries arise from the anterior division of the internal iliac arteries, sweep anterolaterally around the urinary bladder, and ascend in the pre-vesical (retro-pubic) space along the posterior surface of the anterior abdominal wall to the umbilical ring, where they enter the cord alongside the vein (**Figure 1**). The umbilical vein enters the abdomen at the umbilical ring, then courses cranially within the falciform ligament to the left portal vein (portal sinus); a variable fraction of this flow bypasses the hepatic sinusoids via the ductus venosus, which drains into the inferior vena cava.

**Figure 1:**
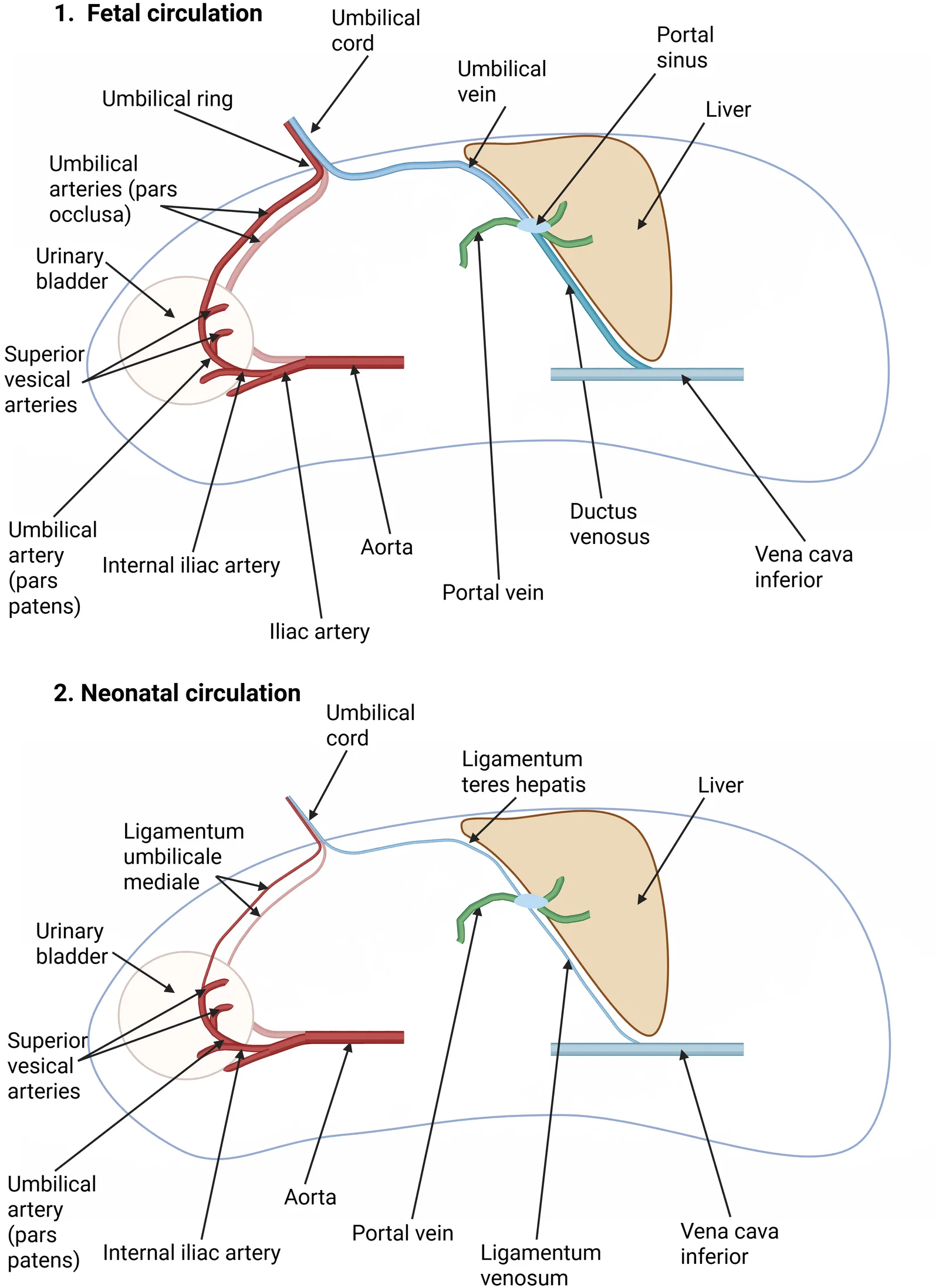
Fetal circulatory system 1) in fetal circulation with patent umbilical vessels and 2) in neonatal circulation with obliterated umbilical vessels (image created with BioRender.com).

At the umbilical ring, the intra-abdominal parts of the vessels become paired as two arteries and one vein and continue extra-abdominally within the umbilical cord toward the placenta. In the extracorporeal portion—the umbilical cord—the paired umbilical arteries wind helically around the single umbilical vein. All three are embedded in Wharton’s jelly, a hyaluronan-rich mucoid connective tissue that cushions the vessels and provides resistance to compression, torsion, and traction. The cord is ensheathed by amnion.

A typical human blood vessel wall comprises three concentric layers arranged from the lumen outward: the tunica interna, media, and adventitia. The tunica interna consists of a single layer of endothelial cells resting on a basement membrane with a thin subendothelial connective layer; in muscular arteries it is bound by an internal elastic lamina. The tunica media contains concentric layers of smooth muscle cells interwoven with elastic fibres and collagen, and in larger muscular arteries may be demarcated externally by an external elastic lamina. The outer tunica adventitia is a connective-tissue sheath and includes vasa vasorum and nervi vasorum in larger vessels.

Umbilical vessels, however, exhibit a distinctive histoarchitecture optimized for rapid postnatal constriction (**Figure 2**). The luminal endothelium contains swollen cells with numerous myoendothelial interdigitations (‘interlocks’) and close contact with the underlying smooth muscle cells. This is supported by an abundance of rough endoplasmic reticulum, a prominent Golgi apparatus, and numerous micro-pinocytic vesicles (Nikolov and Schiebler, 1973; Röckelein et al., 1990). The basement membrane beneath the endothelium is discontinuous with large gaps—or may even be absent—facilitating intimate endothelial-media coupling (Nikolov and Schiebler, 1973; Meyer et al., 1978). In the arteries, the internal elastic lamina is poorly developed or lacking, whereas the vein typically has a well-formed internal elastic lamina but a scant subendothelial connective layer (Spivack, 1946; Nikolov and Schiebler, 1973; Röckelein et al., 1990). The arterial inner tunica media comprises longitudinally oriented smooth-muscle fibres within dense fibrillar connective tissue (Spivack, 1946). These smooth muscle cells are relatively loosely organized, containing sparse myofilament bundles, prominent synthetic organelles, and dilated rough endoplasmic reticulum. The cells are embedded in a metachromatic ground substance that is rich in glycosaminoglycans, particularly chondroitin sulphate, and proteoglycans (Meyer et al., 1978; Nandadasa et al., 2020). The outer tunica media contains better-differentiated smooth-muscle layers arranged largely circumferentially under pressure; during active contraction, these layers assume more oblique orientations in interdigitating groups (Meyer et al., 1978; Röckelein and Scharl, 1988). The intervening extracellular matrix is relatively loose and enriched in heparan sulphate (Spivack, 1946; Meyer et al., 1978; Nandadasa et al., 2020). The vein has a markedly thinner wall and correspondingly lower contractile capacity than the arteries (Nandadasa et al., 2020).

**Figure 2:**
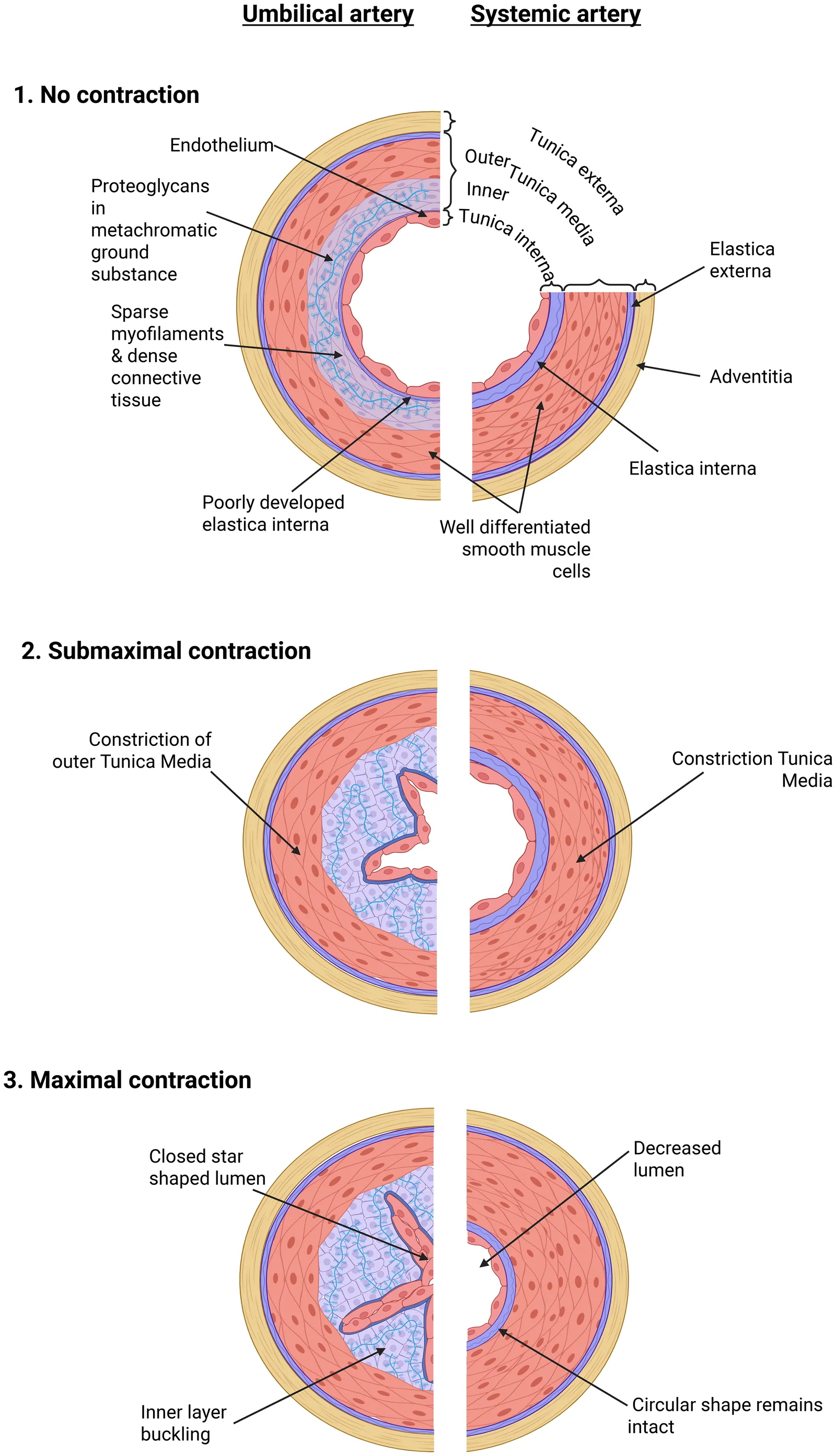
Microscopic comparison between umbilical artery and systemic artery in a constricting vessel (image created with BioRender.com).

## Vasoconstriction physiology

The umbilical arteries constrict in different parts along their length in the umbilical cord, and the local contractions increase in length and number over time. They can be macroscopically seen longitudinally and transversally (**Figure 3**). Early concentric constriction rings form during postnatal physiological umbilical artery closure. These Folds of Hoboken occlude the vessel lumen (Spivack, 1946). In the umbilical vein, only partial occlusion can be seen and is described as “semilunar folds” (Spivack, 1946). These folds lead to dilated parts (Nodules of Hoboken) with entrapped blood where the vessel is stretched and thinned out (Spivack, 1946). In a transversal view of these folds, protrusions of all layers in various shapes and sizes form a star-shaped lumen (**Figure 2**) (Spivack, 1946; Röckelein et al., 1990).

**Figure 3:**
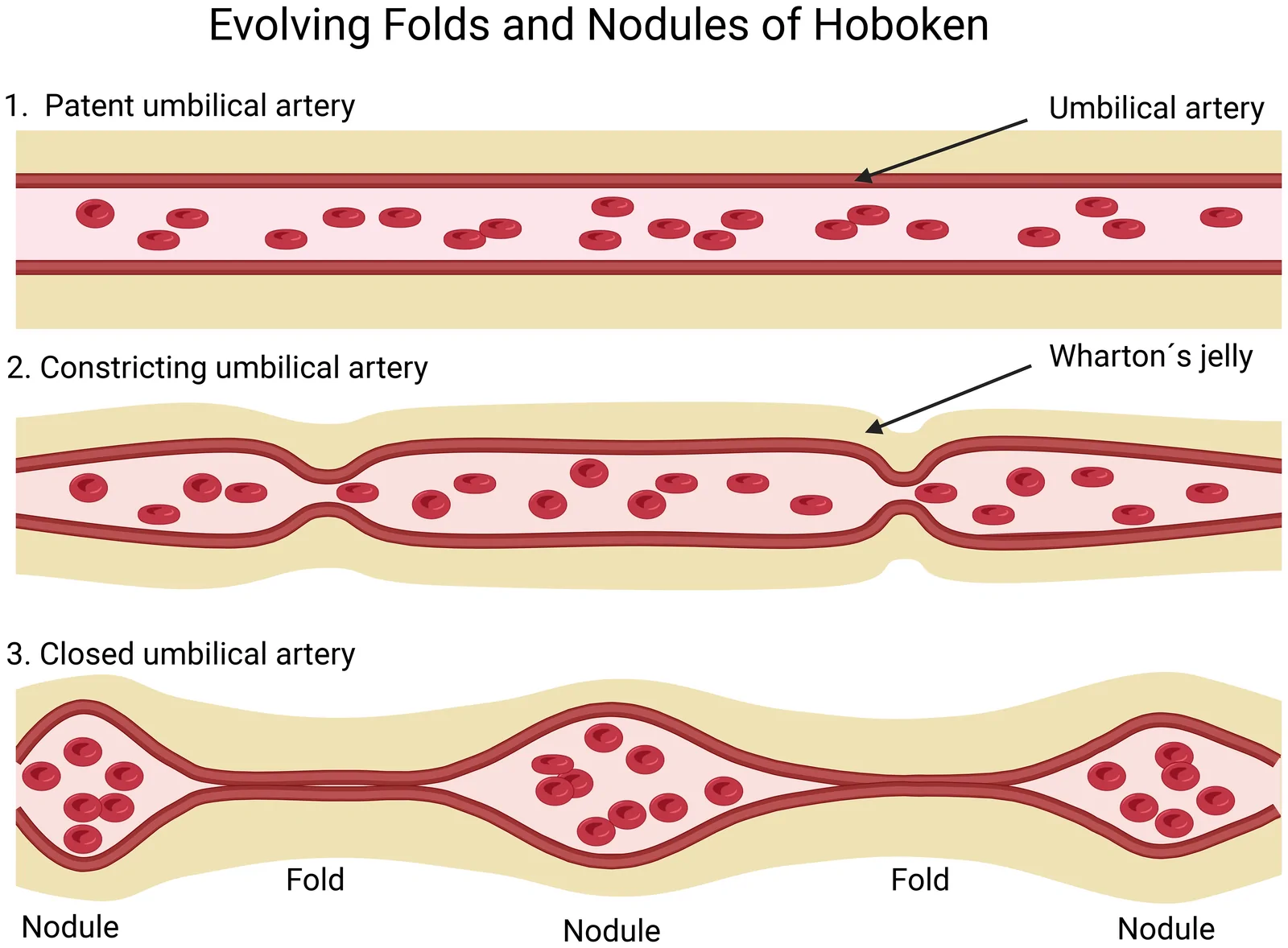
Evolving folds and nodules of hoboken (image created with BioRender.com).

Umbilical vessels enable rapid constriction. The endothelial cellular interlocks, processes, and organelles are developed for cellular connections to quickly pass information from one cell to another and from the endothelium to the tunica media. The absence of different structural elements (e.g. lamina elastica interna) between the endothelial cells and the smooth muscle cells leads to a decreased stability and an increase in intercellular connections for signalling pathways (Nikolov and Schiebler, 1973; Meyer et al., 1978).

The ground substance of the inner layer is rich in glycosaminoglycans, chondroitin sulphate, and proteoglycans, which leads to water adhesion and swelling. During contraction, the volume decreases, which leads to a lower structural stiffness. The circular shape of the vessel is lost, and inner layer buckling occurs (Nandadasa et al., 2020). Thus, the inner layer of the tunica media plays an important role in umbilical vessel closure. It acts as a plastic tissue that is shifted into the lumen. However, it does not constrict on its own and needs to be pushed inward. The small amount of connective tissue in the outer part of the tunica media in comparison to the large amount of smooth muscle cells is crucial for the active vessel closure. The outer part of the tunica media constricts and compresses the inner part over a critical value, where it buckles inward and occludes the lumen (see **Figure 2**).

Notably, differences between intra-abdominal and extra-abdominal vessel closure remain insufficiently characterized. Most studies on umbilical vessel physiology focus on the extra-abdominal part of the umbilical cord, which is easily accessible for research purposes. Consequently, there is a significant scarcity of data concerning the intra-abdominal segments of the umbilical vessels. This knowledge gap is critical because the closure mechanisms and physiological responses of the intra-abdominal portions may differ from those of the cord segment. This has important implications for clinical strategies aimed at prolonging vessel patency for artificial placenta cannulation.

Furthermore, no standardized protocol exists for testing vasoconstriction after exposure to environmental factors or vasoactive agents. While some studies used umbilical rings and tested their response in a solution on an isometric transducer, others used longer segments and quantified the increase in resistance from blood flow (at constant pressure) or blood pressure (at constant flow). Beyond the test system, solutions and environmental conditions also differed between studies.

Consequently, no definitive hierarchy of importance can be established for the vasoactive substances involved in umbilical vessel closure, and extrapolation to the *in vivo* situation remains limited. *In vitro* models permit precise pharmacological characterization but cannot reproduce synergistic *in vivo* effects.

## Mechanical aspects

Mechanical aspects must be considered to further understand umbilical vessel closure. Usually, vasoconstrictors do not lead to complete vessel closure, but to a decreased lumen. However, in the umbilical cord several mechanical factors favour the rapid umbilical vessel closure.

The patency of human vessels depends on an equilibrium between the transmural pressure and the active wall tension (Yao et al., 1977). After birth, the transmural pressure decreases as intravascular pressure drops, while the active wall tension increases due to vasoconstriction of the smooth muscle, together resulting in a complete umbilical vessel closure. The intravascular pressure in the umbilical vessels drops due to the discontinuation of blood flow to the placenta plus a postnatal drop in systemic blood pressure resulting from a blood volume shift caused by the start of pulmonary perfusion. Moreover, pressure gradients across a stenosis (proximal > distal) make distal parts more likely to collapse. It could be hypothesized that the intraabdominal part, especially the umbilical ring, constricts due to neuronal activation, resulting in decreased intravascular pressure in the umbilical cord. In addition, extraluminal pressure increases, because Wharton’s jelly contracts in hypothermic environments promoting vessel closure (Sadler, 2024). Collectively, decreased transmural pressure and increased wall tension lead to umbilical vessel closure.

When considering the histological structure, several factors have to be taken into account. The umbilical vessels are poor in elastin and collagen fibres leading to less stability. This factor gets even more emphasized by the already addressed proteoglycan structure. Thus, higher wall tension results and the vessel is more likely to deform and collapse, especially after the transmural pressure drops. Its passive capabilities to adjust are limited.

In systemic vessels, increase in active wall tension leads to smaller diameters and higher intravascular pressures. Therefore, complete closure of a vessel by vasoconstriction alone is very rare. In umbilical vessels, the two layers of the tunica media play an important role. In terms of the transmural pressure, the inner layer can be seen as the “vessel”, while the outer layer can be seen as the surrounding tissue of the vessel. In this scenario, the extravascular pressure increases due to vasoconstriction and overcomes the intravascular pressure. This results in deformation and collapsing of the umbilical vessel (internal layer).

In summary, vasoconstrictor signalling alone cannot fully explain the rapid and complete occlusion of the lumen. Instead, vasoconstrictors lead to active smooth muscle contraction that must be considered in the context of a mechanically permissive vessel wall. When vasoconstrictive forces exceed a critical threshold, the inner layer buckles. This interaction suggests that biochemical substances must act synergistically with specific macroscopic and microscopic histology in order to convert partial diameter reduction into effective, full vascular closure.

## Physiology of blood vessel flow regulation

Human blood vessels possess the capacity to adapt their diameter to the demands of the circulatory system. This ensures adequate blood flow to various organs, a process that is regulated by multiple mechanisms.

The blood pressure is maintained by adrenergic substances, the renin-angiotensin-aldosterone system, and autonomic nervous innervation. Local perfusion of different organs is ensured by either vasoactive agents or by myoregulation. Vasoactive metabolites like CO_2_, H^+^, and K^+^ dilate specific vessels in skeletal muscle during exercise. Myoregulation occurs in organs that rely on constant perfusion (kidney, brain). Elevated blood pressure increases the vessels’ wall tension and therefore leads to vasoconstriction (Bayliss Effect).

At the cellular level, secretion of vasoactive agents is carried out by neurons, the endothelium, and peripheral organs. The secretion of those agents is the initial phase of the process. These agents are then distributed to their respective sites of action. Their effects include depolarisation, the opening of receptor-based channels, or the activation of receptors in the membrane. This results in an influx of Ca²^+^ from the extracellular space or the sarcoplasmic reticulum. Calcium binds to calmodulin, forming a calcium-calmodulin complex that activates the myosin light chain kinase (MLCK). After this, the kinase then phosphorylates the myosin, resulting in contraction. On the contrary, the myosin light chain phosphatase (MLCP) functions as an antagonist, dephosphorylating the myosin. It has been demonstrated that this phosphatase can be activated by G_s_-coupled receptors (G_s_) in the membrane or by nitric oxide (NO) released from the endothelium. It has been established that both processes result in the formation of cyclic nukleotides, which activate protein kinase (PK) A and G, which in turn activates the MLCP. However, it is important to note that MLCP can also be inhibited by G_q_-coupled receptors (Brandes et al., 2019).

Whether umbilical vessel diameter regulation functions in the same manner as the regulation of other blood vessels needs to be examined. Firstly, neuronal innervation of the umbilical cord was heavily discussed in earlier times (Ellison, 1971; Reilly and Russell, 1977). More recent studies used monoclonal and polyclonal antibodies to neuron associated antigens and showed that there are no neuronal structures in the umbilical cord (Fox and Khong, 1990). However, nervous innervation around the umbilical ring is still discussed and might even regulate the umbilical vein blood flow by constriction of the umbilical ring. Possible effects on umbilical arteries were not studied (Acharya et al., 2006). The effect of innervation of the intraabdominal umbilical vessels and the umbilical ring in umbilical vessel closure must be studied for fully understanding the underlying mechanisms.

Secondly, the umbilical vessels are mainly thought to be regulated autonomously due to vasoactive substances. Many different vasoactive agents induce vasoconstriction. Studies focused on serotonin, prostaglandins and thromboxane, endothelin, acetylcholine, bradykinins, and catecholamines in the human umbilical vessels. Changes in the environmental factors after birth like increased arterial partial pressure of oxygen (pO_2_), lower temperature, and changes in concentrations of the vasoactive substances listed above are being examined in more detail since they are likely related to umbilical vessel closure.

## Environmental changes

### Oxygen

In human umbilical arteries, oxygen induces a concentration-dependent vasoconstriction *in vitro*. The sensitivity of this effect is reduced in environments with a higher pH. At a pH of 7.28 typical for neonatal blood, the threshold for vasoconstriction is at ~ 36 mmHg oxygen partial pressure (McGrath et al., 1986). This supports the vasoconstrictive effect *in vivo*. Oxygen-induced contraction was abolished by prostaglandin inhibitor indomethacin, suggesting prostaglandin-mediated signalling, although the specific prostaglandin involved remained unclear (McGrath et al., 1986; MacLennan et al., 1988).

Other studies, however, refute the hypothesis that oxygen induces vasoconstriction. The physiologic increase in arterial pO_2_ from 27 mmHg to 102 mmHg only led to a minor increase in contraction (5 – 15% of maximum contraction) in 30% of the umbilical arteries (Tuvemo and Strandberg, 1975). This, however small, effect cannot be transferred to an *in vivo* setting. After birth, the arterial pO_2_ rises from 25 – 35 mmHg to 55 – 75 mmHg in 5 to 10 minutes with a wide range (Huch et al., 1973). In the *in vitro* set-up, the partial pressure of oxygen increased within seconds to a higher level and even then the vessel needed up to 15 min to react and constrict (Tuvemo and Strandberg, 1975).

Furthermore, the umbilical vein already carries oxygenated blood *in utero*; oxygen-induced constriction would therefore occur prenatally and compromise fetal oxygen supply.

Oxygen also does not appear to influence the vasoconstrictive force by more energy provision via the electron transport chain and oxidative phosphorylation since inhibition of these factors only leads to a slight decrease in vasoconstriction. In glucose-free media, no vasoconstriction occurs, indicating that anaerobic glycolysis is the main metabolic energy pathway. Therefore, oxygen does not influence vasoconstriction through this mechanism (Nair and Dyer, 1973; Tiritilli, 2000a). However, oxygen plays an important role in the closure of the umbilical artery as a potentiator in other vasoconstrictive pathways (Eltherington et al., 1968).

In summary, oxygen alone is not able to constrict the umbilical arteries sufficiently and is not likely to play any role in the closure of the umbilical vein (Eltherington et al., 1968).

### Temperature

Vessels in the human body can adapt to temperature. Especially skin vessels are important in temperature regulation. They dilate in warm environments to exchange heat, and they constrict in colder environments to keep the core warm.

*In utero*, the umbilical vessels are surrounded by a stable temperature of 37 °C. After birth, the newborn is exposed to the outside temperature. In most cases, this temperature is below 37 °C and therefore the umbilical cord is cooled. Several studies discussed if the umbilical vessels can perform temperature-dependent contractions as well (Sinnathuray, 1968; Boura et al., 1979).

*In vitro*, at an environmental temperature of 22.5 °C and a 40 mmHg partial pressure of oxygen, umbilical cords have a decrease in flow by 70% in the umbilical arteries and by 40% in the umbilical vein. Rewarming leads to vasodilatation, resulting in an increase of flow back to 90%, which shows that temperature affects vasoconstriction rather than other factors (Sinnathuray, 1968).

Boura et al. showed that the temperature-dependent vasospasm was mostly prevented by pretreatment of the umbilical cord, especially the Wharton’s jelly, with indomethacin. It is most likely that a temperature drop causes secretion of prostaglandins which leads to vasoconstriction (Boura et al., 1979). It is still unknown, whether they are produced in the Wharton’s jelly or if the indomethacin injected into the Wharton’s jelly diffused into the vessels’ wall. It remains uncertain, which prostaglandins are produced and via which receptors they induce vasoconstriction.

Cooling the umbilical arteries and vein not only leads to prostaglandin release but also to higher sensitivity for serotonin and acetylcholine. The half maximal effective concentration level (EC_50_) was significantly decreased. However, the maximal effect of those agents was not influenced by the temperature (Atalık et al., 2008).

Not only vasoconstrictive effects occur but also vasodilative responses, albeit less distinctive. In a hypothermic environment, vasodilatation via the NO pathway is lessened in umbilical vessels. NO synthase inhibitors do not influence this decreased vasodilative response. In contrast, guanylate cyclase inhibitors do have an influence (Tiritilli, 2000b; Atalık et al., 2008). The guanylate cyclase might be temperature-dependent and might have a higher activity at higher temperatures.

Whether these findings play a physiologic role must still be discussed. Isolated vessels in these studies got cooled down to 20 – 28 °C (Sinnathuray, 1968; Boura et al., 1979) but no studies measured the umbilical vessel temperature drop after birth *in vivo*. After birth, the umbilical cord is at 37 °C and would need some time to cool down, especially if 37 °C blood runs through the vessels and warms them from inside. In this context, the increased sensitivity to vasoactive agents at lower temperatures is unlikely to be a major driver of umbilical vessel closure, although it may provide a modest additional effect. If prostaglandin biosynthesis occurs in Wharton’s jelly, it could trigger an earlier vasoconstrictive response and thereby contribute physiologically. By contrast, temperature changes are unlikely to induce vasoconstriction in the intraabdominal vessels, as their temperature should remain near 37 °C.

## Vasoactive mediators

### Serotonin

Serotonin (5-hydroxytryptamine, 5-HT) is a potent vasoactive agent released by activated thrombocytes (Brandes et al., 2019). Depending on the effector organ, it induces vasorelaxation via endothelial pathways or vasoconstriction through direct effects on smooth muscle cells.

Activation of endothelial 5-HT-1 receptors increases NO release and promotes vasodilatation (Haugen et al., 1997). In the umbilical vein, 5-HT primarily acts via endothelial 5-HT-1_D_ receptors (Shoeffter et al., 1995) (**Figure 4**), which are inhibitory G protein-coupled receptors. Their beta/gamma subunit activates phosphoinositide 3-kinase and protein kinase B, which then activates the endothelial NO synthase (Gibbs et al., 2018). However, the alpha subunit is involved in vasoconstriction by decreasing cAMP levels and promoting von Willebrand factor (vWF) release, enhancing platelet activation and further 5-HT release (Schlüter and Bohnensack, 1999). In smooth muscle cells, the alpha subunit is also involved in vasoconstriction (Radenković et al., 2009a). In the human umbilical vein, 5-HT-1_B_ receptors contribute to vasoconstriction by reducing intracellular cAMP levels and protein kinase A activity, thereby decreasing myosin light chain phosphatase activity (Rogines-Velo et al., 2002). In the smooth muscle cells, 5-HT-2a receptors mediate concentration-dependent vasoconstriction by increasing Ca^2+^ via Gq-coupled signalling (Radenković et al., 2009a).

**Figure 4:**
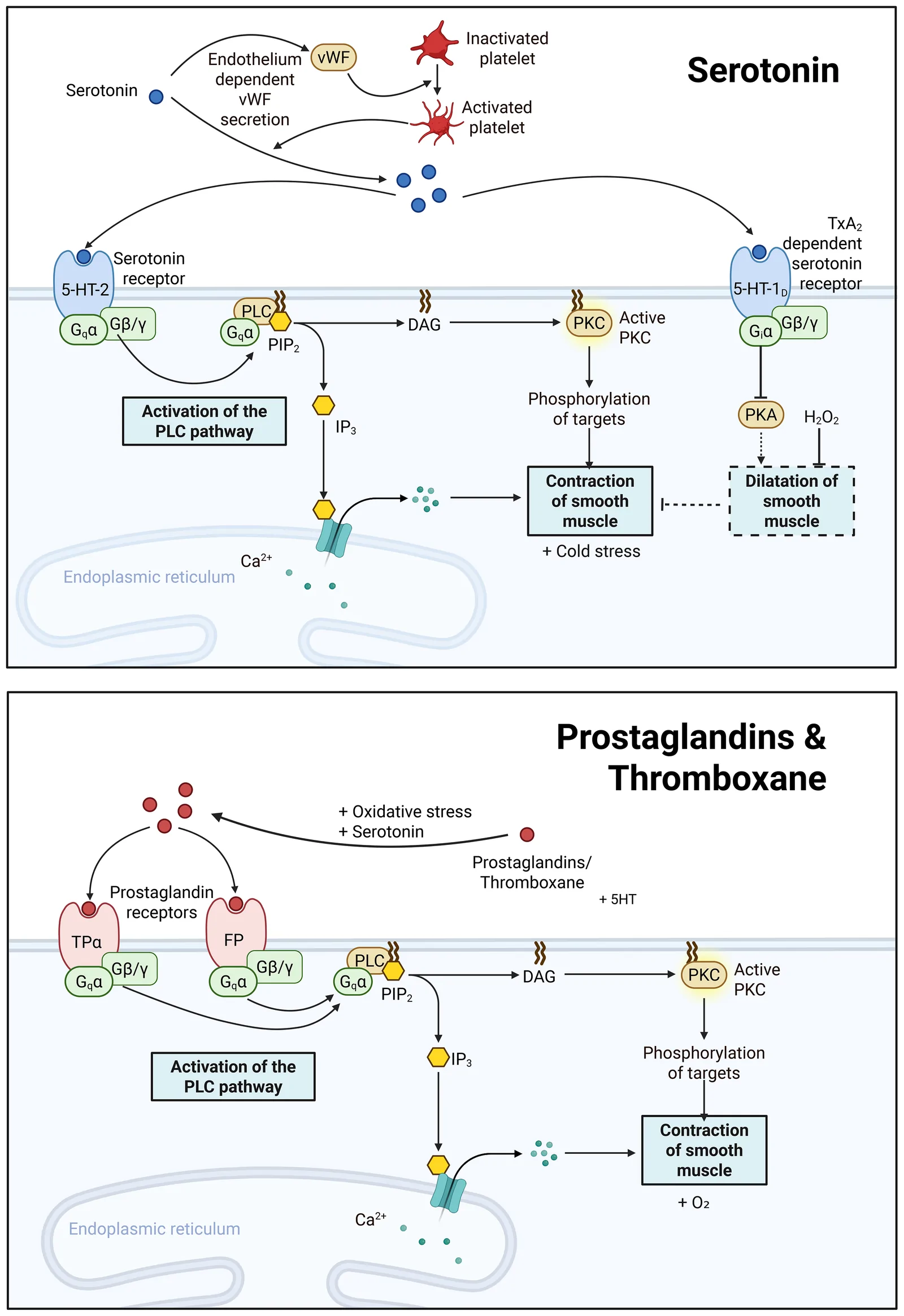
Serotonin-, prostaglandin- and thromboxane-dependent vasoconstriction in umbilical smooth muscle cells (image created with BioRender.com). 5-HT-2 & 5-HT-1_D_, G protein-coupled serotonin receptors; G_q_α, Gβ/γ, α, β, and γ subunit; DAG, Diacylglyceride; FP, G protein-coupled prostaglandin F receptor; G_q_α, Gβ/γ, α, β, and γ subunit; H_2_O_2_, Hydrogen peroxide; IP_3_, Inositol trisphosphate; PIP_2_, Phosphatidylinositol-4,5-bisphosphate; PKA, Protein kinase A; PKC, Protein kinase C; PLC, Phospholipase C; TPα, G protein-coupled thromboxane A2 receptor; G_q_α, Gβ/γ: α, β, and γ subunit, TxA_2_, Thromboxane A_2_; vWF, von Willebrand factor.

The response of the umbilical vessels to 5-HT depends on the localization of the different receptors. The affinity of endothelial 5-HT-1 receptors is higher than of muscle cell 5-HT-2 receptors (Andrade et al., 2025). Therefore, at low 5-HT concentrations, endothelial 5-HT-1 receptor activation predominates, leading to vasodilatation, whereas higher 5-HT concentrations activate 5-HT-2 receptors and induces vasoconstriction in smooth muscle cells (Haugen et al., 1997). This may reflect stronger signalling or higher receptor density on smooth muscle cells than on the endothelium.

After birth, the umbilical vessels constrict because of several changes in the 5-HT pathway. The concentration of 5-HT in the umbilical cord increases from 135 ± 52 ng/mL up to 199 ± 62 ng/mL (Berman, 1966). During this rise in concentration, a biphasic response occurs. A short, initial vasodilatation is followed by a more sustained vasoconstriction. Endothelium-dependent vasodilatation seems to be faster and shorter in duration than smooth muscle-mediated vasoconstriction via 5-HT-2 receptors.

Postnatal vasoconstriction is further enhanced by increased sensitivity to 5-HT. Hypothermia, increased oxygen tension, and oxidative stress potentiate its effects. Hypothermia increases sensitivity without changing the maximal response (Atalık et al., 2008). Rising arterial pO2 after birth increases the maximal contraction force induced by 5-HT (Nair and Dyer, 1973). However, this oxygen dependency is limited. At physiologic levels, 5-HT leads to 30% more contraction force than under hyperoxic conditions (Quan et al., 2003). Under hypoxic conditions, prostacyclin secretion reduces contraction, since cyclooxygenase blockade by indomethacin does not reduce the vasoconstrictive force while endothelial NO synthase (eNOS) inhibition does (Tiritilli, 2000a).

Other authors claim that cyclooxygenase activity is increased at high oxygen levels, promoting TxA_2_ release, which activates functional 5-HT-1 receptors in smooth muscle cells leading to contraction (MacLennan et al., 1989; Templeton et al., 1991). This receptor would explain the higher contraction force at lower 5-HT concentrations in oxygen-rich environment since the affinity to 5-HT-1 receptors is higher than to 5-HT-2 receptors.

Other studies confirm the TxA_2_ dependency of 5-HT-mediated contractions. Inhibition of TxA_2_ reduces endothelium-independently the sensitivity of 5-HT, particularly under physiological pO_2_ and 5-HT conditions (Cruz et al., 1997). The increase in maximum contraction is logarithmically related to pO_2_, with small increases causing large effects (Nair and Dyer, 1973). Umbilical arteries show greater oxygen-dependence than veins (Nair and Dyer, 1972).

Higher oxidative stress potentiates vasoconstriction. After birth more oxidative stress occurs (Moore et al., 2019) Hydrogen peroxide inhibits endothelium-dependent vasodilatation by inhibition of the eNOS pathway (Watanabe et al., 1996), although its specific effect on 5-HT-1-mediated vasodilatation remains uncertain.

In summary, 5-HT is a very potent vasoconstrictor with an increase in contractile force due to postnatal increases in concentration combined with increased sensitivity mediated by oxygen tension, temperature, and oxidative stress.

### Thromboxane A_2_

Thromboxane A_2_ (TxA_2_) is a vasoactive eicosanoid produced primarily by thrombocytes and endothelial cells. It acts mainly on G_q_-coupled thromboxane receptors (TPα) on smooth muscle cells, inducing vasoconstriction (**Figure 4**), and enhances thrombocyte activation and degranulation.

In human umbilical arteries, TxA_2_ is produced in the tunica media (Benedetto et al., 1984; Neri Serneri et al., 1983). After birth, levels of its stable metabolite TxB_2_ increase from 100 pg/mL up to 250 pg/mL (Mitchell et al., 1978), partly due to increased oxidative stress. Peroxides stimulate the cyclooxygenase (COX) and lead to increased TxA_2_ secretion (Walsh et al., 1993). In addition, 5-HT stimulates TxA_2_ release (Bjøro, 1986).

Besides its role as a second messenger in oxygen-dependent 5-HT-mediated vasoconstriction, TxA_2_ itself is a very potent vasoconstrictor. At physiological levels (117 mmHg pO_2_ and 1 nmol/L concentration), human umbilical arteries constricted with about 2.2 g contraction force, approximately half the contraction force of 5-HT (4.5 g) (Quan et al., 2003).

### Prostaglandins

Prostaglandins (PGs) are eicosanoids which can be produced in nearly every tissue. PGG_2_ is cyclooxygenase-dependently produced from arachidonic acid and converted by peroxidases to PGH_2_, which is then converted into prostaglandins such as PGE_2_, PGF_2α_, PGD_2_, PGI_2_ (prostacyclin), by specific prostaglandin synthases, and into TxA_2_ by thromboxane synthase. They play an important role in neonatal adaptation. After birth, the PG levels decrease and cause closure of the ductus arteriosus (Olley and Coceani, 1981).

In the umbilical cord, PG levels seem to increase due to birth stress, since higher concentrations were observed in vaginal deliveries compared to C-sections (Jonsson et al., 1976). Activity of PG endoperoxide-H synthase, the key enzyme for prostaglandin synthesis, increases before and during labour (Teixeira et al., 1994), leading to increased PGH_2_ concentration, which is predominantly converted to prostacyclin (Bjøro et al., 1986).

PGs act via different G protein-coupled PG receptors and can be either vasodilators or vasoconstrictors depending on receptor subtype. While some PGs are exclusively vasoconstrictive (e.g. PGF_2α_) or vasodilative (e.g. PGI_2_), some can induce both reactions (PGE_2_).

In umbilical arteries, vasoconstrictive effects of PGE_2_ and PGF_2α_ were abolished by TP receptor antagonists (Boersma et al., 1999), indicating the absence of functional prostaglandin E (EP) and prostaglandin F (FP) receptors. In the umbilical vein, however, TP and FP receptors are expressed (Daray et al., 2003; Errasti et al., 2009) (**Figure 4**). The FP receptor works similarly to the TP receptor, since they are both G_q_ coupled.

PGE_2_ and PGF_2α_ are about equipotent and induce concentration-dependent vasoconstriction. The response to PGE_2_ was significantly higher at high oxygen levels (102 mmHg) than at *in utero* conditions (27 mmHg) (Tuvemo and Strandberg, 1975). Thus, postnatal PG release and oxygen-enhanced PG sensitivity can promote vasoconstriction. However, underlying mechanisms remain unclear, especially since EP1 and EP2 receptors are not expressed and therefore not involved. Other studies suggest that PGF_2α_ is twice as potent as PGE_2_ (Adaikan and Karim, 1974). This discrepancy between the studies might reflect differences in oxygen tension or temperature.

In contrast, PGE_1_ exhibits biphasic effects. In low concentrations (< 10 µg/mL), PGE_1_ dilates the vessel, while at higher levels (> 15 µg/mL) it leads to vasoconstriction (Tuvemo and Strandberg, 1975). More studies are needed regarding the receptors, second messengers, and environmental factors involved in this vascular response.

### Endothelin

Endothelin (ET) is a peptide hormone with three isoforms (ET-1, ET-2, and ET-3), which is produced by the endothelium and acts as a strong vasoconstrictor. ET binds to G_q_-coupled ET_A_ and ET_B_ receptors on smooth muscle cells (**Figure 5**). Endothelial ET_B_ receptors are also G_i_-coupled, acting as constriction antagonist by mediating NO production.

**Figure 5:**
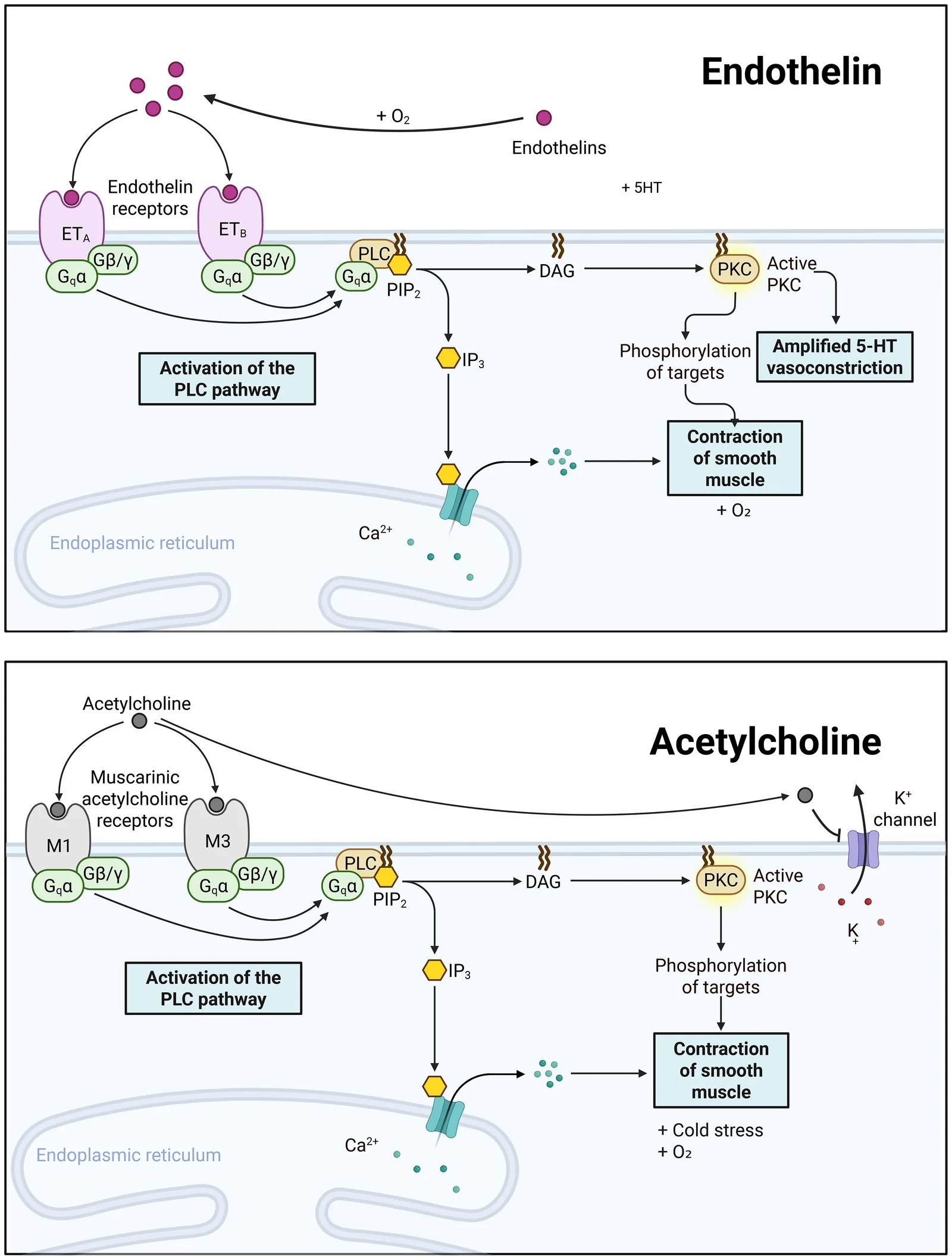
Endothelin- and Acetylcholine-dependent vasoconstriction (image created with BioRender.com). 5-HT, Serotonin; ET_A_, ET_B_, G protein-coupled endothelin receptors; G_q_α, Gβ/γ, α, β, and γ subunit; DAG, Diacylglyceride; IP_3_, Inositol trisphosphate; M1, M3, G protein-coupled muscarinic acetylcholine receptor; G_q_α, Gβ/γ, α, β, and γ subunit; PIP_2_, Phosphatidylinositol-4,5-bisphosphate; PKC, Protein kinase C; PLC, Phospholipase C.

In the umbilical cord, ET is found especially in amnion cells around the cord, partly in Wharton’s jelly, and umbilical vein endothelium (Salamonsen et al., 1992). After the first breath, arterial plasma ET concentrations increase approximately sixfold independent on birth method, even if ET had been absent before (Nisell et al., 1990). This likely occurs due to ET diffusion from Wharton’s jelly or amnion cells or from systemic production (e.g. in placenta).

ET-1 is the most potent isoform, while ET-3 only has an effect in the umbilical vein. The precursor Big-ET is less potent but still vasoconstrictive. Big-ET-1 is more potent than Big-ET-2 in the umbilical artery, both are equivalent in the umbilical vein, and Big-ET-3 is ineffective in both vessels (Rizzi et al., 1998).

ET-1 induces concentration-dependent vasoconstriction, starting at 100 pmol/L, which is the estimated mean concentration after delivery in the umbilical artery (Haegerstrand et al., 1989; Nisell et al., 1990). With increasing oxygen tension, the effect increases (Bodelsson et al., 1996).

Although this suggests that ET plays a role in umbilical vessel closure, more recent studies report no significant contraction at physiological levels (117 mmHg pO_2_ and 10–100 pmol/L ET), with thresholds at 3–30 nmol/L (Quan et al., 2003). Nevertheless, subthreshold ET-1 concentrations enhance 5-HT-induced vasoconstriction, likely via G_q_-coupled ET receptors and intracellular protein kinase C cascade, since protein kinase C inhibition abolished the response (Okatani et al., 1995). This amplifying effect occurred at 250 pmol/L, which is above concentrations measured in plasma. Intracellular concentration and the *in vivo* situation might differ.

In summary, ET is a strong vasoactive agent, whose concentration increases after birth, and oxygen potentiates its effect. However, the concentrations are allegedly too low for direct vasoconstriction. Instead, ET likely amplifies 5-HT-dependent vasoconstriction.

### Acetylcholine

Acetylcholine (ACh) is a neurotransmitter of the parasympathetic and somatic nervous systems. Although it usually induces vasodilatation in peripheral vessels via muscarinic receptors, it induces vasoconstriction in umbilical vessels. Muscarinic ACh receptors, most likely the G_q_-coupled M1 and M3 subtypes, mediate this effect (Pujol Lereis et al., 2006) (**Figure 5**). Inhibition of protein kinase C and calcium inhibitors reduce the contraction.

Additionally, potassium currents and large-conductance calcium-activated potassium channel activity in smooth muscle cells are reduced (Chen et al., 2015; Zhu et al., 2016), limiting repolarization and maintaining depolarization and therefore constriction. This vasoconstriction is not dependent on the endothelium but attenuated by 5-HT receptor antagonist methysergide (Yoshikawa and Chiba, 1991).

After birth, umbilical cord temperature decreases. At 28 °C, ACh sensitivity is significantly higher than at 37 °C. However, maximal response is unchanged. This effect is independent of endothelial NO synthase (Atalık et al., 2008).

After birth, rising oxygen saturation of the umbilical artery also induces vasoconstriction that can be abolished by atropine, a muscarinic antagonist. Although ACh might be secreted paracrinally, it has not been detected in the umbilical cord (Welsch and Wenger, 1980), possibly due to fast hydrolysis. Alternatively, atropine may inhibit other factors (e.g. TxA_2_) via unknown cross-reactions. This question remains unsolved and needs further studies.

### Bradykinin

Bradykinin is a vasoactive peptide, generated by plasma or tissue kallikreins. Its precursors are high- and low-molecular weight kininogens. Plasma kallikreins hydrolyse the high-molecular weight kininogens via the coagulation cascade, while tissue kallikrein activates low-molecular weight kininogens (Leeb-Lundberg et al., 2005).

In umbilical blood, bradykinin levels rise sixfold after birth due to decreased temperature, increased oxygen tension, and granulocyte exposure (Melmon et al., 1968). Bradykinin interacts with G_q_-coupled B1 and B2 receptors, also in the umbilical vessels (Gobeil et al., 1996), inducing intracellular Ca^2+^ influx. However, in umbilical vessels, B1 receptor inhibition does not alter vascular tone, whereas B2 receptor activation produces concentration-dependent contraction (**Figure 6**). This contraction is modulated by cyclooxygenase, more precisely TxA_2_ which enhances the contractile effect (Radenković et al., 2007).

**Figure 6:**
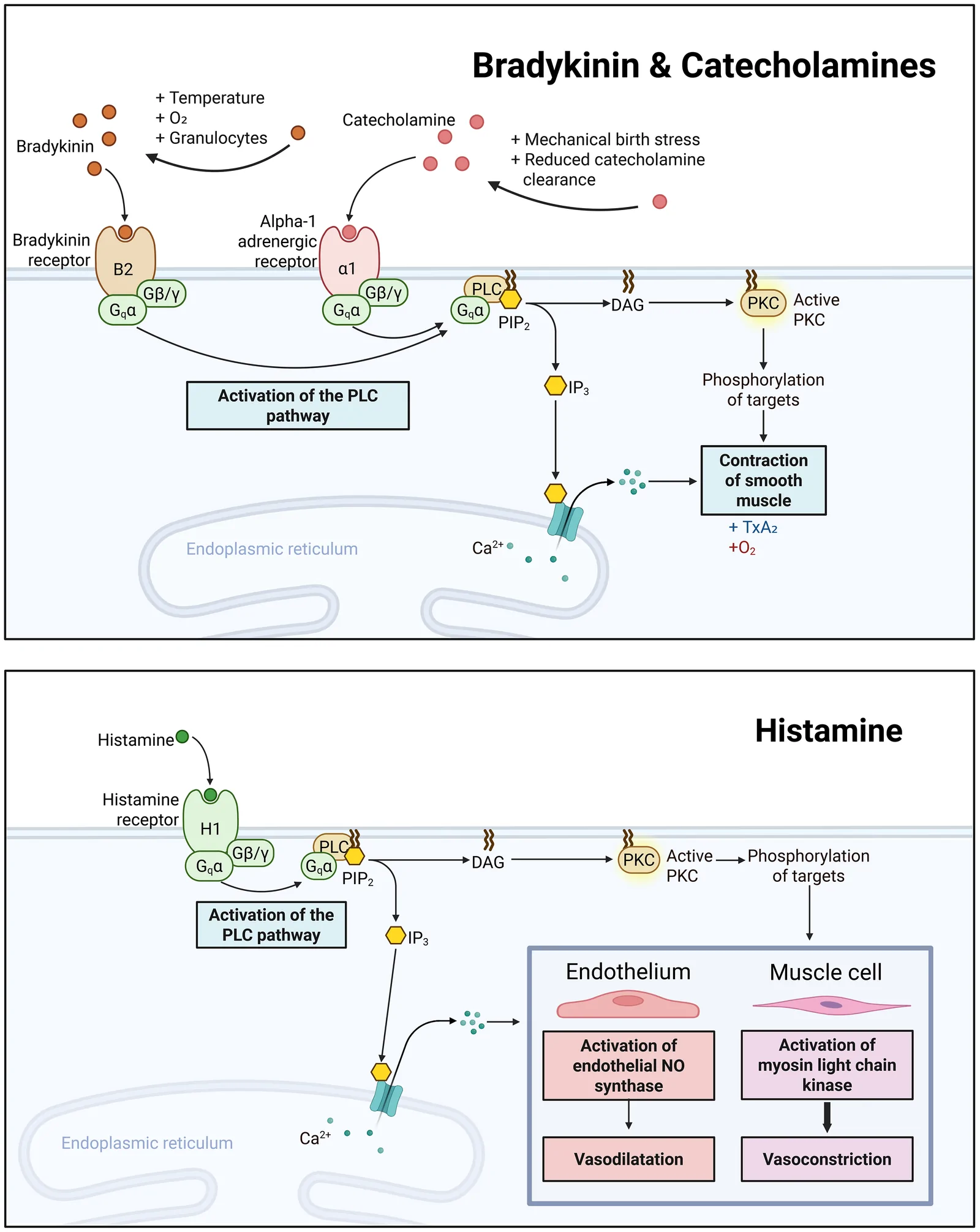
Bradykinin-, catecholamine-, and histamine-dependent vasoconstriction (image created with BioRender.com). α_1_, G protein-coupled adrenergic receptors; G_q_α, Gβ/γ, α, β, and γ subunits; B2, G protein-coupled bradykinin receptors; DAG, Diacylglyceride; H1, G protein-coupled histamine receptors; IP_3_, Inositol trisphosphate; PIP_2_, Phosphatidylinositol-4,5-bisphosphate; PKC, Protein kinase C; PLC, Phospholipase C; TxA_2_, Thromboxane A_2_.

Oxygen leads to sensitization: at high oxygen levels, less bradykinin is required for sufficient vasoconstriction responses, while at low oxygen levels, only high concentrations are effective (Oberhänsli-Weiss et al., 1972; Eltherington et al., 1968).

In a more recent study, the bradykinin response showed tachyphylaxis (Quan et al., 2003). It is still uncertain whether this effect does happen in *in vivo* conditions as well. However, bradykinin might play a role in the initial phase of the vasoconstriction.

### Catecholamines

Catecholamines are neurotransmitters with various functions. In human vessels, adrenaline and noradrenaline act on α-adrenoceptors, triggering a G_q_-coupled cascade that causes vasoconstriction (**Figure 6**). Catecholamines are usually secreted by neurons or the adrenal medulla during stress.

After birth, catecholamine levels in the umbilical artery increase to 62 nmol/L (Lagercrantz and Bistoletti, 1977). This increase is based on two factors. Firstly, due to birth stress, catecholamines get secreted; hypoxia and mechanical stress during vaginal birth lead to higher catecholamine levels than C-sections (Wang and Zhang, 2009). Secondly, catecholamine clearence is reduced; *in utero*, the placenta contributes substantially to clearance, and this mechanism is lost after birth (Stein et al., 1993). After birth, this mechanism is missing.

Catecholamines most likely interact with α-adrenoceptors since β-adrenoceptor anatgonists do not alter catecholamine-induced vasoconstriction (Eltherington et al., 1968). Additionally, the vasoconstrictive effect of noradrenaline increases at higher postnatal oxygen levels (Meirelles et al., 1977).

Overall, the effect is very low in comparison to other vasoactive agents. In perfusion experiments, only a slight increase in perfusion pressure occured, meaning the vasoconstriction force of catecholamines is limited (Yoshikawa and Chiba, 1991). However, they might have a supportive effect.

### Histamine

Histamine is a biological amine mainly involved in anaphylactic reactions, secreted by mast cells and typically inducing vasodilatation via H1 receptors.

In umbilical vessels, histamine can cause both vasodilatation and vasoconstriction (**Figure 6**). Vasodilatation occurs in mid-term (18–22 weeks) umbilical cords through H1 receptors on endothelial cells, activating phospholipase C, Ca^2+^ influx, and therefore eNOS. This relaxes smooth muscle due to NO (Izumi et al., 1995).

At full term, histamine induces vasoconstriction (Izumi et al., 1995; Quan et al., 2003), possibly via H1 receptors on smooth muscle cells, though the mechanism remains unclear. Its *in vivo* role in umbilical vessel closure is uncertain, as plasma histamine levels could not be measured (Mitchell and Porter, 1970).

## Impaired vasorelaxation

The vascular tone is usually regulated by vasoconstrictive and vasodilative factors. It was already discussed that vasoconstrictive agents and factors lead to umbilical vessel closure. Moreover, impaired vasorelaxation plays an important role as well. The most important vasorelaxant in human vessels is the endothelium-derived relaxing factor, nitric oxide (NO). NO activates, on the one hand, the soluble guanylate cyclase in the smooth muscle cells, which results in increased cGMP levels and activation of protein kinase G. This kinase activates the MLCP and inhibits Ca^2+^ channels. On the other hand, NO activates potassium channels, decreasing the membrane potential and therefore hindering cellular depolarization (Lovren and Triggle, 2000). Depolarization of the smooth muscle cells would result in Ca^2+^ influx and vasoconstriction. Due to those factors, NO is a very potent vasodilator.

NO is synthesized in the endothelium by the endothelial NO synthase. In umbilical vessels, there is only a small percentage of NO synthase-immunoreactive endothelial cells reducing their capability to produce NO (Sexton et al., 1995).

Not only is the total amount of NO production lower than in other vessels, but also its effect. Pre-contracted umbilical vessels were not able to dilate more than 30% after treatment with several agonists of the NO pathway (e.g. NO, NO donor, guanylate cyclase activator) (Provitera et al., 2019).

Umbilical vessels differ from other human vessels. Cyclic guanosine monophosphate (cGMP) usually leads to vasodilatation. In umbilical arteries, the cGMP levels increase after treatment with vasoconstrictors, raising the possibility that these vasoconstrictive pathways require cGMP as a second messenger, especially in oxygen-rich environment, when the activity of the guanylate cyclase increases (Clyman et al., 1975a).

Nevertheless, it is more likely that these cGMP levels are higher because of a negative feedback mechanism. The guanylate cyclase gets activated to start a cascade acting as a counterpart for the strong vasoconstriction. However, usually this vasodilative response is not strong enough and umbilical vessel constriction and closure occurs.

The cAMP levels do not increase after vasoconstrictives are applied. Thus, the negative feedback mechanism is linked to only the guanylate cyclase and the cGMP levels (Clyman et al., 1975b).

The increase in oxygen tension after birth has no influence on the vasodilative response after treatment with vasodilators (Leung et al., 2006) but the total amount of NO changes. The endothelial NO synthase (eNOS) is oxygen-dependent. In the umbilical artery, hypoxia leads to an increase in eNOS activity. In contrast, umbilical vein endothelial cells decrease the eNOS activity in hypoxic conditions (Krause et al., 2012). After birth, the umbilical arteries have higher oxygen levels than *in utero*, thus, leading to lower eNOS activity, and the umbilical vein has lower oxygen levels in the early stages of birth which also results in lower eNOS activity.

In summary, umbilical vessels have an impaired vasorelaxation due to a small amount of NO synthase-immunoreactive cells. They produce less NO, especially after birth when the oxygen content in the vessels is changing and the NO is less effective.

## Hierachy of vasoconstrictive forces

According to the current literature, a variety of environmental factors and vasoactive substances may be involved in umbilical vessel closure. However, the significance and relevance of these factors differs. While some factors act as primary drivers, others act as amplifiers or secondary modulators. We propose that the primary drivers of vasoconstriction are 5-HT and TxA2, which induce strong and sustained contractions. Amplifiers such as changes in oxygen tension and temperature, and endothelin, enhance the sensitivity and magnitude of these primary drivers. Secondary modulators such as acetylcholine, bradykinin, catecholamines and histamine appear to have only limited significance. They are more likely to support or modulate contraction than act as dominant vasoconstrictors. This information is very important for maintaining vessel patency and improving cannulation feasibility. This redundancy implies that several factors need to be addressed. The primary drivers must be modulated simultaneously. Antagonists of the local 5-HT-2, 5-HT-1_D_ and TPα receptors could minimise harmful side effects. Furthermore, environmental factors such as temperature and humidity should be maintained as they are in the womb. Modulation of the secondary modulators may not be sufficient, so further research should focus mainly on the primary drivers and amplifiers.

## From pregnancy complications to cannulation feasibility: determinants of umbilical vessel patency

The physiological closure of the umbilical arteries and vein after birth is a complex process that can be significantly influenced by various pathological conditions and perinatal events. This knowledge is of critical importance for the development of artificial placenta technology and the establishment of large bore vascular access via umbilical vessel cannulation in extremely premature or compromised neonates. The target population for artificial placenta therapy consists almost exclusively of neonates with significant pathology—infants who are often growth-restricted, premature, acidotic, or born to mothers with complex hypertensive or metabolic disorders. Consequently, the “window of opportunity” for cannulation—the time between delivery and irreversible vessel spasm or closure—is highly variable and pathology-dependent. Notably, the basic mechanisms of umbilical vessel closure of the extra-abdominal parts in the cord in healthy term neonates are increasingly well understood. However, the literature specifically addressing how fetal and pregnancy pathologies affect the timing and quality of postnatal umbilical vessel closure remains remarkably scarce. Most available studies focus on antenatal vascular reactivity, structural changes, or Doppler hemodynamics rather than on the actual closure dynamics after birth. This gap in knowledge represents an important area for future research, particularly given the clinical implications for successful umbilical vessel cannulation and extracorporeal life support.

In preterm infants, the umbilical artery smooth muscle cells are not fully differentiated, exhibiting a lower content of contractile proteins like smoothelin compared to term infants, which may lead to less efficient vasoconstriction and potentially a wider cannulation window (Roffino et al., 2012). However, this must be balanced against the structural immaturity and fragility of preterm vessels.

Hypertensive disorders of pregnancy, such as preeclampsia, create a hyper-reactive and structurally remodelled vascular environment that poses significant challenges for large bore cannulation. Umbilical arteries from preeclamptic pregnancies show an augmented contractile response to serotonin and dysfunction in ATP-sensitive potassium (K_ATP_) channels, leading to a more depolarized, spasm-prone state (Zampieri et al., 2024). This is compounded by placental oxidative stress, which increases the production of lipid peroxides and the potent vasoconstrictor thromboxane, while decreasing the vasodilator prostacyclin. This creates a pro-constrictive milieu that may accelerate vessel closure and narrow the cannulation window (Walsh et al., 1993; Walsh et al., 1996). Histomorphometric analysis further reveals that umbilical arteries in preeclampsia have a significantly larger vessel wall area and a higher wall-to-lumen ratio. This indicates hypertrophy that can stiffen the vessel and make mechanical dilation for cannula insertion more difficult (Blanco et al., 2011).

Maternal diabetes mellitus is associated with distinct structural and functional vascular anomalies that directly impact cannulation feasibility. Neonates of mothers with gestational diabetes are more likely to have hyper-coiled umbilical cords, which can complicate the threading of cannulas and may predispose the vessel to kinking during extracorporeal circulation (Ezimokhai et al., 2001). However, this structure only occurs in the extra-abdominal part. This can make the first few centimetres of cannulation difficult and would require more flexible catheters. Histological examination shows degeneration of smooth muscle fibres and endothelial disruption, suggesting vessel fragility that increases the risk of dissection during cannulation (Tenaw Goshu, 2022). The vascular reactivity is also altered, with enhanced acetylcholine-induced vasoconstriction via epigenetic mechanisms (He et al., 2025), and some studies suggesting a reduced contractile response to serotonin but a modified response to bradykinin (Radenković et al., 2007; Radenković et al., 2009b).

In cases of chorioamnionitis, bacterial exotoxins, such as those from Group B *Streptococcus*, can directly induce umbilical vein contraction, independent of host inflammatory mediators, potentially causing pathogen-induced spasm that compromises vessel patency (Hyde et al., 1989). The associated fetal inflammatory response often leads to vasculitis and fibrin deposition within the umbilical vessels, increasing the risk of thrombosis and potential embolic events during cannulation (Ducey et al., 2014).

Meconium-stained amniotic fluid (MSAF) exerts a biphasic effect on umbilical vessel tone that is highly relevant to cannulation timing. In the acute phase, meconium components including bile acids and ET-1 induce potent vasoconstriction, with studies demonstrating contractile responses comparable to 63% of maximal 5-HT-induced contraction (Altshuler and Hyde, 1989). However, with prolonged exposure (> 12 hours), meconium causes meconium-associated vascular necrosis (MAVN), characterized by smooth muscle cell death, resulting in flaccid, dilated vessels that fail to close normally after birth (Sienko, 1999; Cimic and Baergen, 2016). For cannulation, this presents a paradox: acute MSAF creates severe spasm with risk of intimal tear during dilation, while chronic MSAF/MAVN produces vessels that lack the tone to grip the catheter, increasing the risk of leakage and dislodgement.

Fetal growth restriction (FGR) is characterized by structural alterations in the umbilical cord and decreased NO-induced relaxation of the umbilical vein (Peyter et al., 2014), as well as increased hemodynamic resistance in the umbilical arteries, often seen as absent or reversed end-diastolic flow on Doppler ultrasound, indicating a tonically constricted or stiff vessel that may be challenging to cannulate (Acharya et al., 2005). FGR is also associated with abnormal cord morphology, such as hypocoiling, and thinner Wharton’s jelly, which may reduce the cord’s ability to resist external compression during manipulation and increase susceptibility to meconium-induced damage (Wan et al., 2025).

The mode of delivery also plays a role in determining the initial vascular tone at the time of potential cannulation. Infants delivered vaginally have significantly higher levels of catecholamines, which promote vasoconstriction, compared to those delivered by elective caesarean section, where placenta-to-infant transfusion may also be affected (Kleinberg et al., 1975; Vogl et al., 2006). This suggests that vessels in vaginally delivered infants may exhibit higher initial tone and faster closure, potentially narrowing the cannulation window compared to those from elective caesarean sections. Furthermore, delayed cord clamping can lead to a transient state of acidosis as metabolites wash out from peripheral tissues, which may alter the sensitivity of the vessel wall to vasoactive agents and affect the response to pharmacological interventions aimed at maintaining vessel patency (Wiberg et al., 2008). Thus, the timing and efficiency of postnatal umbilical vessel closure are not uniform but are heavily modulated by antenatal pathology, with direct implications for artificial placenta cannulation strategies. Preeclampsia is characterized by hypertrophic, hyper-reactive vessels prone to powerful spasm mediated by 5-HT and TxA_2_, potentially requiring rapid cannulation and aggressive anti-spasmodic therapy. Diabetes presents structural challenges with hypercoiling and vessel fragility, necessitating careful cannula selection and insertion technique. FGR is associated with high-resistance, hypocoiled vessels that may already be tonically constricted. Chorioamnionitis introduces the risk of pathogen-induced contraction and luminal thrombosis, requiring consideration of anticoagulation strategies. MSAF presents a time-dependent challenge, with acute exposure causing severe spasm and chronic exposure leading to structurally incompetent vessels requiring gentle handling and alternative securing techniques. The mode of delivery and the degree of perinatal acidosis further contribute to the variability in vessel closure dynamics. Understanding these specific pathological phenotypes is essential for developing tailored cannulation protocols that anticipate the structural and functional hurdles presented by each condition, ultimately optimizing the success of artificial placenta therapy and large bore umbilical vascular access in the most vulnerable neonatal population.

## Summary

The closure of the umbilical vessels after birth is a rapid, robust, and multifactorial process. It is driven by the abrupt transition from the intrauterine to the extrauterine environment. While essential to prevent neonatal blood loss, this closure represents a major barrier to artificial placenta support, which requires timely large-bore cannulation of the umbilical arteries and vein.

Several postnatal changes promote vasoconstriction and increase the sensitivity of umbilical vessels to constrictor mediators:

- Temperature drop
- Increase in oxygen tension
- Loss of placental vasodilator influence
- Mechanical stimulation

In addition, vasoconstrictor pathways with multiple mediators contribute to umbilical vessel constriction, creating redundancy and synergy:

- 5-HT
- TxA_2_
- ET-1
- Other vasoactive agents

Furthermore, anatomical and histological mechanisms support the umbilical vessel constriction.

Beyond active smooth-muscle contraction, the umbilical arteries exhibit structural features that facilitate rapid closure. The arterial tunica media is functionally bilayered: an inner proteoglycan-rich layer predisposes to inward buckling and luminal occlusion, while an outer smooth-muscle-rich layer actively constricts. Longitudinal intimal protrusions (Folds of Hoboken) further promote mechanical occlusion of the lumen (**Figure 7**).

**Figure 7:**
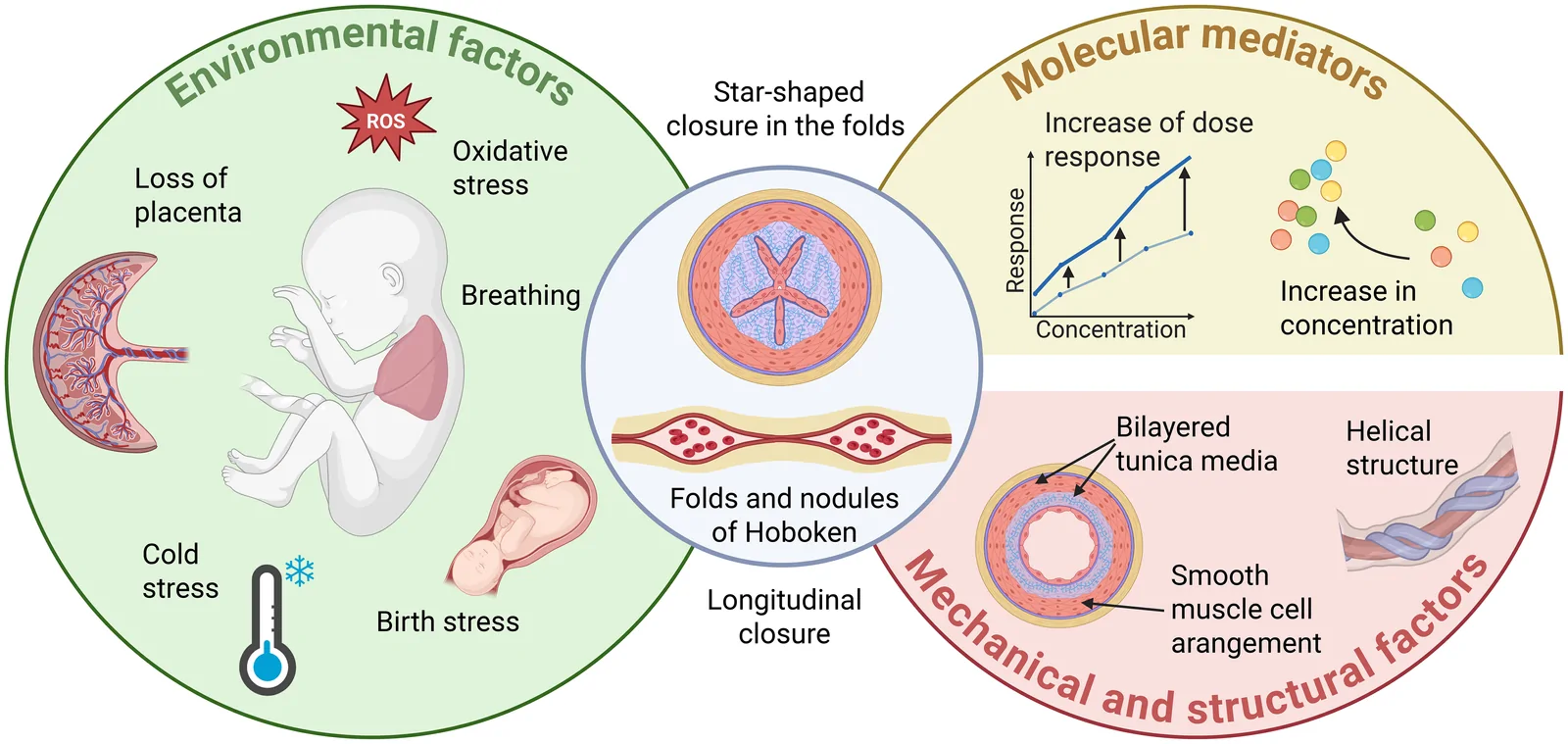
Environmental factors, molecular mediators and mechanical and structural factors contributing to umbilical vessel closure (image created with BioRender.com). ROS, reactive oxygen species.

These physiological processes and anatomical or histological conditions vary depending on various perinatal comorbidities, such as infants with growth restriction, premature infants, acidotic infants, or infants born to mothers with complex hypertensive or metabolic disorders.

It should be noted, however, that the underlying literature consists largely of older ex vivo studies of extra-abdominal umbilical artery rings. Therefore, further *in vivo* studies are needed to better quantify individual factors and mechanisms, thereby enabling a translation into postnatal physiology and providing a basis for intervening in this physiology within the context of cannulation for an artificial placenta. Closure of the intra-abdominal segments in particular requires further study, since constriction of the umbilical arteries must be prevented during artificial placenta treatment. Research should further establish the hierarchy of the individual vasoconstrictive factors *in vivo*, as it remains uncertain whether umbilical blood pressure and flow during treatment are sufficient to overcome their combined effect. Targeted approaches, such as selective inhibition of the receptor subunits discussed above, should therefore be evaluated to vasodilate the umbilical vessels and thereby improve cannulation feasibility and extracorporeal blood flow.

## Conclusion

Umbilical vessel closure is therefore not a single pathway phenomenon but a physiological program combining triggers, mediator cascades, and a “fail-safe” structural design. Maintaining vessel patency for artificial placenta support will likely require a multi-pronged strategy that simultaneously (i) reduces external triggers, (ii) suppresses dominant vasoconstrictor pathways, and (iii) mechanically preserves lumen geometry long enough to achieve safe large-bore cannulation. However, it is important to note that the vast majority of existing research has focused on *in-vitro* extra-abdominal (cord) segments of the umbilical vessels. Consequently, a significant knowledge gap remains concerning the specific physiological and structural closure mechanisms of the intra-abdominal umbilical vessels, which may have critical implications for developing effective patency-prolonging strategies necessary for artificial placenta applications.
